# Increasing the power of genome wide association studies in natural populations using repeated measures – evaluation and implementation

**DOI:** 10.1111/2041-210X.12535

**Published:** 2016-02-05

**Authors:** Lars Rönnegård, S. Eryn McFarlane, Arild Husby, Takeshi Kawakami, Hans Ellegren, Anna Qvarnström

**Affiliations:** ^1^ Department of Clinical SciencesSwedish University of Agricultural SciencesSE‐75007UppsalaSweden; ^2^ Department of Animal EcologyEvolutionary Biology Centre (EBC)Uppsala UniversityNorbyvägen 18DSE‐75236UppsalaSweden; ^3^ Department of BiosciencesMetapopulation Research CentreUniversity of HelsinkiPO Box 65FI‐00014HelsinkiFinland; ^4^ Department of BiologyCentre for Biodiversity DynamicsNorwegian University of Science and TechnologyN‐7491TrondheimNorway; ^5^ Department of Evolutionary BiologyEvolutionary Biology Centre (EBC)Uppsala UniversityNorbyvägen 18DSE‐75236UppsalaSweden

**Keywords:** *Ficedula albicollis*, genomic relationship, hierarchical generalized linear model, single‐nucleotide polymorphisms

## Abstract

1. Genomewide association studies (GWAS) enable detailed dissections of the genetic basis for organisms' ability to adapt to a changing environment. In long‐term studies of natural populations, individuals are often marked at one point in their life and then repeatedly recaptured. It is therefore essential that a method for GWAS includes the process of repeated sampling. In a GWAS, the effects of thousands of single‐nucleotide polymorphisms (SNPs) need to be fitted and any model development is constrained by the computational requirements. A method is therefore required that can fit a highly hierarchical model and at the same time is computationally fast enough to be useful.
2. Our method fits fixed SNP effects in a linear mixed model that can include both random polygenic effects and permanent environmental effects. In this way, the model can correct for population structure and model repeated measures. The covariance structure of the linear mixed model is first estimated and subsequently used in a generalized least squares setting to fit the SNP effects. The method was evaluated in a simulation study based on observed genotypes from a long‐term study of collared flycatchers in Sweden.
3. The method we present here was successful in estimating permanent environmental effects from simulated repeated measures data. Additionally, we found that especially for variable phenotypes having large variation between years, the repeated measurements model has a substantial increase in power compared to a model using average phenotypes as a response.
4. The method is available in the r package RepeatABEL. It increases the power in GWAS having repeated measures, especially for long‐term studies of natural populations, and the R implementation is expected to facilitate modelling of longitudinal data for studies of both animal and human populations.

## Introduction

The fast development of molecular genetic techniques offers novel integration possibilities by making it feasible to investigate how processes at the molecular genetic level relate to processes at the different phenotypic levels, that is ranging from developmental pathways up to morphological and behavioural traits. Genomewide association studies (GWAS) that link molecular genetic information with phenotypic information have successfully been applied to detect causal mutations and to understand the genetic architecture of complex traits in both plants and animals, including humans (Rosenberg *et al*. 2010; Flint & Eskin 2012). Dissection of the genetic architecture of adaptive traits is essential in understanding evolutionary processes and can be used to infer past processes as well as future predictions of adaptation. For these reasons, evolutionary biologists are interested in applying these techniques to their own study systems, and as the previously prohibitive price of genotyping has come down, are becoming able to do so (Slate *et al*. 2010; Ellegren 2014). Thus, it has become feasible to study evolution at the genomic level in a wide range of organisms (Slate *et al*. 2010; Ekblom & Galindo 2011). For example, recent studies have applied genomic data, and more traditional selection analyses using pedigree information, to explain the maintenance of variation in Soay sheep horn shape (Johnston *et al*. 2013), and in collared flycatcher clutch size (Husby *et al*. 2015), where life‐history trade‐offs appear to be involved in maintaining genetic variation at one or several loci in both species.

Long‐term monitoring studies in wild animals include repeated measures on individuals and allow for estimation of year effects, age effect, senescence and changes over time (Clutton‐Brock & Sheldon 2010). For example, collared flycatchers (*Ficedula albicollis*), which are the subject of a number of long‐term studies (e.g. Qvarnström, Rice & Ellegren 2010), are philopatric (Pärt 1994), with high recapture rates (Pärt & Gustafsson, 1989), and sometimes live to be more than 5 years old (Gustafsson & Pärt 1990). Collared flycatchers have been used to study a wide range of central evolutionary questions including senescence (Gustafsson & Pärt 1990), effects of climate change (Both *et al*. 2004; Robinson *et al*. 2012), sexual selection (Qvarnström, Pärt & Sheldon 2000; Qvarnström, Brommer & Gustafsson 2006) and microevolution (Merilä, Kruuk & Sheldon 2001). Since they hybridize with the closely related pied flycatcher (*Ficedula hypoleuca*), they are also used as a model for speciation research (Sætre et al., 1997; Sæther et al., 2007; Qvarnström *et al*. 2009; Ellegren *et al*. 2012; Qvarnström *et al*. 2015). The extensive use of collared flycatchers as an avian model for ecological and evolutionary research was the main reason for the development of a 50 L illumina iSelect SNP array for GWAS analysis of this species (Kawakami *et al*. 2014). Repeated measures of the same individuals are not exclusive to studies of flycatchers, but is a shared feature among wild animal GWAS programs, such as Soay sheep (Hayward *et al*. 2013), great tits (Husby, Visser & Kruuk 2011) and bighorn sheep (Martin *et al*. 2014).

Repeated measures are difficult to model using traditional gwas software as the most frequently used software have not been primarily developed for studies on wild populations where repeated observations are common and the number of observation per individual varies. Thus, several methods to solve this problem have been applied. A first possibility is to use the average phenotypic value for each individual, which might be appropriate if the data are balanced with equal number of observations per individual. Furthermore, important ecological cofactors that change over time for an individual, such as age and year, cannot be included when average phenotypes are used. An alternative to using mean estimates is to compute a linear mixed model not including the SNP effect and subsequently use the fitted random effect for each individual as response in an ordinary least squares analysis (Johnston *et al*. 2011; Santure *et al*. 2013).

However, this approximate method reduces power and inflates false positives, if error estimates are not also carried into the next analysis (Postma 2006; Valdar *et al*. 2009; Hadfield *et al*. 2010; Ekine *et al*. 2013). The user may alternatively resort to non‐standard gwas software fitting a linear mixed model at each marker including both a random polygenic effect and a random permanent environmental effect, where the former adjusts for the relatedness between individuals and the latter for repeated observations on each individual. This is possible using the commercial software asreml (Gilmour *et al*. 2009) for instance, but would be time‐consuming and to our knowledge has not been applied to large GWAS. Consequently, in many statistical analyses of GWAS, repeated measurements are treated as a burden rather than an asset, and thus, there is a need to develop a method and software for genomewide association analyses on populations having repeated measurements on related individuals.

Here, we develop a user‐friendly statistical method that can be used for GWAS where there are related individuals that may have repeated observations. The method also gives flexible modelling of random effects (including spatial correlations) for GWAS in general. The method assumes Gaussian phenotypes, and we further investigated the adequacy of the method for use with binary data. In a recent paper, Husby *et al*. (2015) applied the method on clutch size in collared flycatchers and here we assess an open‐source implementation of the method in the RepeatABEL package. Although the focus of this paper is on applications of GWAS in natural populations, we expect that the RepeatABEL package will be useful in human studies where methods to perform GWAS on longitudinal data has also been recently investigated and discussed (Beyene & Hamid 2014). The RepeatABEL package is available on CRAN (https://cran.r-project.org) and is part of the GenABEL suite of packages at http://www.genabel.org.

## Materials and methods

### Statistical Methods

The statistical model used in GWAS can be described in terms of a linear regression fitted at every marker position on the genome, where the phenotype is modelled as response and the marker dosage as covariate. For a SNP, the marker dosage can be graded as 0, 1 or 2, according to the number of non‐reference alleles that an individual is carrying. The basic model applied assumes that the residuals in this regression model can be treated as independent having a common variance. If the individuals are related, this assumption is violated and as a result the computed significance for the fitted SNP effects will be inflated. Several computational tools for GWAS have been developed and two of the most commonly used by biologists are PLINK (Purcell *et al*. 2007) and GenABEL (Aulchenko *et al*. 2007b). As GenABEL is implemented in the freely available statistical software r (http://www.r-project.org), it is also fairly user‐friendly. Yu *et al*. (2006) suggested that confounding effects in GWAS caused by individuals being related should be modelled using a linear mixed model including a random polygenic effect. If this effect is excluded, a GWAS will produce inflated $-\log_{10}P$ values. The reason for this is two‐fold. Closely related individuals tend to share common environmental effects as well as a common genetic background (Flint & Eskin 2012). Methods to correct for individuals being related have been developed and implemented in the widely used GenABEL package.

In studies having repeated observations, the $-\log_{10}P$ values will also be inflated because observations from the same individual can be correlated. In studies with repeated measures, permanent environmental effects that the individual is exposed to throughout its lifetime can be substantial, but methods to correct for repeated observations have not been widely developed in standard gwas software. To address this problem, we developed a method for GWAS that includes related individuals having repeated observations. The method is implemented in the r package RepeatABEL and is GenABEL‐dependent which makes it user‐friendly especially for those acquainted with the GenABEL package. Although the focus of the package is on models for related individuals having repeated observations, it can be used for linear models having arbitrary covariance structures.

The standard model for testing the significance of a SNP effect can be formulated as a linear model

$$y=\mu +x_{snp}\beta_{snp}+e$$

where *μ* is an intercept term, $x_{snp}$ is the SNP dosage, $\beta_{snp}$ is the SNP effect, and the residuals *e* are assumed independent coming from a common normal distribution. The model is fitted for each marker location along the genome, and a standard Wald statistic can be used to compute the *P*‐value for each SNP. It is also possible to include other cofactors as fixed effects in the model (e.g. sex, age and year), but to simplify the notation only the intercept and SNP effects are included in the following equations.

A linear mixed model including also random polygenic effects, *g*, and permanent environmental effects, *p*, is

(eqn 1) $$y=\mu +x_{snp}\beta_{snp}+Zg+Zp+\epsilon$$

where *Z* is an incidence matrix relating the individuals to their observed values. The random effects are multivariate Gaussian

$$\begin{array}{c} g∼N(0,\sigma_{g}^{2}G)\\ p∼N(0,\sigma_{p}^{2}I_{n})\\ \epsilon ∼N(0,\sigma_{\epsilon }^{2}I_{N}) \end{array}$$

where *G* is the genomic relationship matrix (VanRaden 2008), *I* is the identity matrix with subscript indicating its size, and *n* and *N* are the number of individuals and total number of observations, respectively. The same model can be written as

(eqn 2) $$y=\mu +x_{snp}\beta_{snp}+e$$

e∼N(0,V)

where $V=ZGZ^{\prime }\sigma_{g}^{2}+ZZ^{\prime }\sigma_{p}^{2}+I_{N}\sigma_{\epsilon }^{2}$.

#### Fitting algorithm

The *P*‐value for the SNP effect in model (1) can be computed from the ratio of the estimate of $\beta_{snp}$ and its standard error, that is a standard Wald test statistic, which is equivalent to using the test statistic

$$z_{1}=\frac{x_{snp}^{\prime }V^{-1}\left(y-\mu \right)}{\sqrt{x_{snp}^{\prime }V^{-1}x_{snp}}}$$

Here $z_{1}$ is asymptotically standard normal, where *V* and *μ* are computed at the estimated values of the variance components.

An alternative is to use the score test statistic

$$z_{2}=\frac{x_{snp}^{\prime }V_{0}^{-1}\left(y-\mu_{0}\right)}{\sqrt{x_{snp}^{\prime }V_{0}^{-1}x_{snp}}}$$

which is also asymptotically standard normal but here $V_{0}$ and $\mu_{0}$ are computed from a model where the SNP effect $x_{snp}\beta_{snp}$ has been excluded. A computational advantage of using a score test is that the *P*‐values can be computed without having to estimate $\beta_{snp}$, but on the other hand the estimates of $\beta_{snp}$ needs to be computed separately if required. The score test is implemented in the mmscore function in GenABEL for instance (Aulchenko *et al*. 2007b).

The following test statistic approximates the Wald and score tests

$$z_{3}=\frac{x_{snp}^{\prime }V_{0}^{-1}\left(y-\tilde{\mu }\right)}{\sqrt{x_{snp}^{\prime }V_{0}^{-1}x_{snp}}}$$

where $\tilde{\mu }$ is the estimated intercept term and is computed for each SNP from a model in the generalized least squares

(eqn 3) $$y=\mu +x_{snp}\beta_{snp}+e$$

$$e∼N(0,V_{0}\sigma_{e}^{2}).$$

##### Algorithm

The statistic $z_{3}$ is computed for each SNP in the RepeatABEL package to obtain *P*‐values. It is calculated in a computationally efficient way together with an estimate for the SNP effect ${\hat{\beta }}_{snp}$. The algorithm works as follows:

1. Fit the model *y* = *μ *+ *Zg* + *Zp* + *ε* to estimate $V_{0}$.
2. For each SNP fit, the generalized least squares $y=\mu +x_{snp}\beta_{snp}+e$ with $e∼N(0,V_{0}\sigma_{e}^{2})$. This can be performed in a computationally efficient way:
  - Eigen decompose $V_{0}=\Gamma \Lambda \Gamma^{\prime }$
  - Rotate the linear model to obtain a new, but equivalent, model having independent residuals by pre‐multiplying the left‐ and right‐hand sides with $\Lambda \Gamma^{-0.5}$
  - Estimate *μ* and *β* using ordinary least squares and subsequently compute the sum of squared residuals to get the *P*‐value using a Wald test for the null hypothesis $H_{0}:\beta_{snp}=0$.

Note that the eigen decomposition only needs to be performed once and that QR‐factorization (Golub & Van Loan 2012) is used to solve the ordinary least squares to speed up the computations. One may also note that the algorithm works for any estimated $V_{0}$ and can be applied to arbitrary covariance structures, and by setting $V_{0}$ equal to the identity matrix the model reduces to ordinary linear regression. The hglm package in r (Rönnegard, Shen & Alam 2010) is suitable for estimating random effects and covariance structures and is used in the RepeatABEL package for variance component estimation for model (3).

### Simulations

#### Data sets

The flycatcher data set includes 10 000 autosomal marker genotypes from 849 collared flycatchers (*Ficedula albicollis*) on chromosomes 1–24. 1118 collared flycatchers from the Swedish island of Öland (56^∘^44′N 16^∘^40′E) were genotyped on an Illumina iSelect BeadChip (Kawakami *et al*. 2014). These birds are part of a long‐term monitoring project, where we caught, ringed and sampled blood from breeding adults and their offspring in the population (Qvarnström *et al*. 2009). A total of 50 000 SNPs were included on the chip, of these, 45 138 were successful (Kawakami *et al*. 2014). A total of 2400 SNPs were not unambiguously placed on a scaffold during genome assembly, and 2083 SNPs were excluded because the genotypes of these SNPs were not unambiguously determined due to poor genotype quality (Kawakami *et al*. 2014; Husby *et al*. 2015). We removed SNPs that did not pass our quality control for a call rate of at least 95%, minor allele frequency of at least 0·01 and a *P*‐value of Hardy Weinberg equilibrium more than 0·001. This left us with 38 598 SNPs for analysis, of which we have chosen 10 000 for the simulations. Some of the individuals were highly related and the off‐diagonal elements in the genetic relationship matrix had values up to 0·64. The data set is included in the RepeatABEL package as a GenABEL object ‘flycatchers’.

#### Simulated SNP effects and phenotypes

For each scenario, a QTL effect, completely linked to an arbitrary SNP, was simulated. One or several QTLs were simulated along the genome, and their location was sampled at random among all SNPs.

The phenotypes were either simulated as Gaussian or binary (0/1). For a Gaussian trait, *y* was computed as $y=x_{snp}\beta_{snp}+Zg+Zp+e$, with *g*,* p* and *e* sampled from normal distributions having zero means and variances $\sigma_{g}^{2}$, $\sigma_{p}^{2}$ and $\sigma_{e}^{2}$, respectively. The simulated phenotypes were computed using the simulate_PhenData function in the RepeatABEL package.

The binary trait values were simulated using a threshold model where the underlying phenotype, $y_{u}$, was simulated as a Gaussian trait described above. The observed phenotype, *y*, was subsequently given a value *y* = 1 for $y_{u}>\tau$ and *y* = 0 otherwise, where *τ* is a given threshold.

If otherwise not stated, the data were simulated using the flycatcher data and with random effects having variance components of $\sigma_{g}^{2}=1$, $\sigma_{p}^{2}=1$, and $\sigma_{e}^{2}=1$, together with a fixed additive SNP effect of 0·5 (i.e. the difference between homozygotes is 1·0).

Two main data structures were simulated. In the balanced scenario, each individual had two observations, and in the unbalanced scenario, the number of observations per individual was on average 2·0 with a variance of 2·0.

## Results

### Comparison of the Repeated Measurements Model to a Model with Individual Averages as Response

We evaluated the advantage of using the repeated measurements model compared to a model using individual averages as response by simulating 20 replicates under different scenarios. For a balanced data scenario, where individuals having two observations each were simulated, no apparent differences in effect size estimates (Table 1) nor $-\log_{10}P$‐values (Table 2) were found. In an unbalanced scenario without year effects, where the number of observations per individual was on average 2·0 with a variance of 2·0 there was a slight (2%) improvement in $-\log_{10}P$‐values for the simulated QTL. The flycatcher genotype data that we used for the simulation study included 849 individuals and 10 000 markers.

**Table 1:** Average (SD) of estimates from repeated measurement model (rGLS) compared to a model fitting average phenotypes as response (using the mmscore function in GenABEL)^a^

Balanced	Year effects^b^	Method	$\hat{\beta }{}_{snp}$	$h^{2}$	$pe^{2}$	*λ*
Yes	No	mmscore	0·484 (0·14)	0·392 (0·06)	NA	1·01 (0·01)
Yes	No	rGLS	0·484 (0·14)	0·340 (0·04)	0·334 (0·03)	1·01 (0·01)
Yes	Yes	mmscore	0·476 (0·07)	0·424 (0·06)	NA	1·01 (0·01)
Yes	Yes	rGLS	0·475 (0·07)	0·362 (0·03)	0·321 (0·03)	1·00 (0·01)
No	No	mmscore	0·475 (0·08)	0·366 (0·08)	NA	1·00 (0·01)
No	No	rGLS	0·474 (0·08)	0·339 (0·05)	0·329 (0·05)	1·00 (0·01)
No	Yes	mmscore	0·477 (0·17)	0·233 (0·07)	NA	1·00 (0·00)
No	Yes	rGLS	0·466 (0·10)	0·357 (0·04)	0·313 (0·04)	1·01 (0·01)

a Simulated $\beta_{snp}\;=\;0\cdot 5$, heritability $h^{2}\;=\;\frac{1}{3}$, coefficient of permanent env. effects $pe^{2}\;=\;\frac{1}{3}$.

b Simulated year effect explaining 57% of the total phenotypic variance. Year effects fitted as fixed effects in the repeated measurement model.

**Table 2:** Comparison of *P*‐values between methods: repeated measurement model (using rGLS) vs. a model fitting average phenotypes as response (using mmscore)^a^

Balanced	Year effects^b^	Increase in $-\log_{10}P$‐values^c^	Correlation of $-\log_{10}P$‐values
Yes	No	1·0%	0·9999
Yes	Yes	0·8%	0·9999
No	No	**1·7%**	0·9948
No	Yes	**40%**	0·8319

a Simulated $\beta_{snp}\;=\;0\cdot 5$, additional polygenic heritability $h^{2}\;=\;\frac{1}{3}$, coefficient of permanent env. effects $pe^{2}\;=\;\frac{1}{3}$.

b Simulated year effect explaining 57% of the total phenotypic variance. Year effects fitted as fixed effects in the repeated measurement model.

c At the simulated QTL. Significant differences between methods shown as bold text.

**Figure 1:**
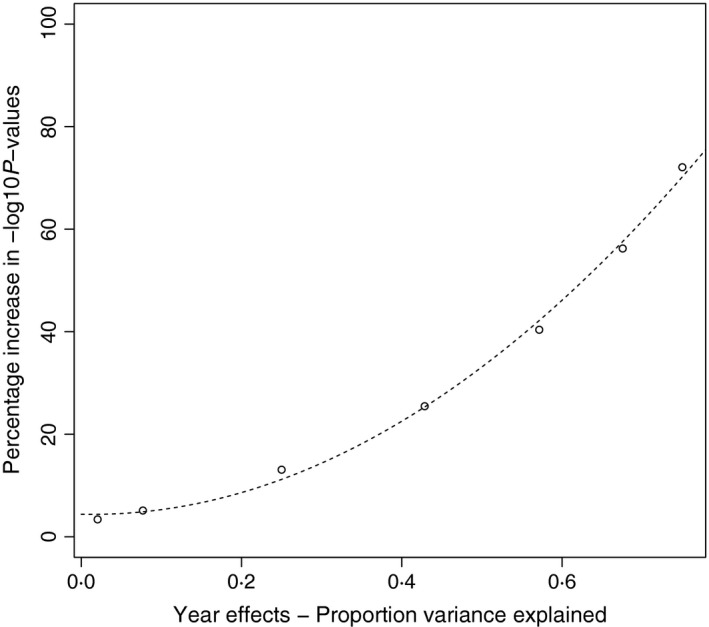
Percentage increase in $-\log_{10}P$‐values depending on the size of the year effects. Flycatcher data were used to simulate a population with unbalanced number of observations per individual.

For an unbalanced scenario including year effects, the two methods deviated substantially. This was expected because large yearly variations cannot be captured in a model using individual averages, whilst the repeated measurements model explicitly models the year effects to reduce the noise in the data and increase the SNP effect signal. The improvement in $-\log_{10}P$‐values for the simulated QTL increased with the variance explained by the year effects (Fig. 1). For example, for a trait like tarsus length in collared flycatchers where the year effects explain a bit over 7% of the total phenotypic variance, we found a 5% increase in $-\log_{10}P$‐values, when using the repeated measures model compared to the individual averages model.

When we simulated a trait that was influenced by year effects explaining 25% of the total phenotypic variance, we found a $-\log_{10}P$‐value of 5·4 from the individual averages model. This can be contrasted with the $-\log_{10}P$‐value of 6·1 that we found using the repeated measurements model. This increase in power is indeed large and corresponds to increasing the number of individuals of around 15% in a standard GWAS.

### Further Assessment of the Repeated Measurements Model

The performance of the repeated measures method was further evaluated using simulations based on the flycatcher genotype data with four observations per individual and 100 simulation replicates per scenario.

The proportion of false positives at a specific QTL position was close to 5%, at a 95% significance level, when no QTL effects were simulated (Table 3). Furthermore, the inflation factor *λ* was close to 1·00, and it was concluded that the repeated measurement model performed as expected.

**Table 3:** Performance of repeated measurement model for different number of SNP effects and effect sizes

No. QTL	$\beta_{snp}$	${\hat{\beta }}_{snp}$	Average *P*	Prop. $P<10^{-6}$	Estimated variance components			*λ*
					Polygenic	Perm. env.	Residual	
0	0	0·00 (0·11)	0·50 ( 0·29)	0	1·03 (0·10)	0·98 (0·08)	1·00 (0·03)	1·00 (0·01)
1	1·0	1·00 (0·10)	<0·01 (<0·01)	0·97	1·30 (0·16)	1·06 (0·10)	1·00 (0·03)	1·02 (0·02)
1	1·5	1·50 (0·10)	<0·01 (<0·01)	0·98	1·66 (0·27)	1·14 (0·15)	1·00 (0·03)	1·01 (0·02)
20	1·0	1·00 (0·21)^a^	0·01 (0·05)	0·75	6·00 (0·54)	2·58 (0·26)	0·99 (0·03)	1·21 (0·02)

a Average over the 20 estimated effects.

The estimated SNP effects were close to the simulated ones under all tested scenarios. When a large number of QTL effects were simulated, however, the estimated genetic variance component was inflated, because part of the simulated QTL effects were picked up as genetic variance in the model fitting each SNP separately. The inflation factors *λ* were also substantially greater than 1·00 under this rather extreme scenario with 20 simulated QTLs having large effects.

For the flycatcher data, where several individuals are highly related, the two variance components $\sigma_{g}^{2}$ and $\sigma_{p}^{2}$ could be separated (Table 4), although the variance component for the permanent environmental effect, $\sigma_{p}^{2}$, absorbed a small proportion (8%) of the genetic variance when no permanent environmental effect was simulated. For populations having a large proportion of closely related individuals, the need to model the two variance components separately increases, whilst in a population of less related individuals it might be sufficient to only include $\sigma_{g}^{2}$ in the model. In a population of less related individuals, ${\hat{\sigma }}_{g}^{2}$ is expected to absorb most of the permanent environmental effects and thereby produce correct *P*‐values for the SNP effects. However, in our proposed method it is easy to include both variance components and is suggested as the default model.

**Table 4:** Performance of estimated variance components in the repeated measurement model. Two scenarios simulated including permanent environmental effects in the simulations (with a variance of 1), and without permanent environmental effects simulated

Perm. env. effect simulated	Estimated variance components			*λ*
	Polygenic	Perm. env.	Residual	
Yes	1·03 (0·10)	0·98 (0·08)	1·00 (0·03)	1·00 (0·01)
No	0·90 (0·08)	0·08 (0·03)	0·99 (0·03)	1·00 (0·01)

### Binary Phenotypes

For the simulated binary phenotype data (Table 5), the proportion of false positives was close to 5% as expected when no QTL effects were simulated. The estimated variance components add up to 0·25 as expected for a binary proportion of 0·5. Furthermore, the estimated genetic variance was close to the expected value of 0·053 (see Appendix S1) except for the very extreme scenario of 20 major QTL effects.

**Table 5:** Performance of repeated measurement model for binomial data. Using an underlying Gaussian distribution, an equal binary proportion of zeros and ones was simulated for individuals having the common allele

No. QTL	$\beta_{snp}$	${\hat{\beta }}_{snp}$	Average *P*	Prop. $P<10^{-6}$	Estimated variance components			*λ*
					Polygenic	Perm. env.	Residual	
0	0	0·00 (0·03)	0·50 ( 0·29)	0	0·06 (0·01)	0·06 (0·01)	0·13 (0·01)	1·00 (0·01)
1	1·0	0·22 (0·02)	<0·01 (<0·01)	0·96	0·06 (0·01)	0·06 (0·01)	0·13 (0·01)	1·02 (0·02)
1	1·5	0·31 (0·02)	<0·01 (<0·01)	0·98	0·07 (0·01)	0·06 (0·07)	0·12 (0·01)	1·02 (0·02)
20	1·0	0·13 (0·03)	0·01 ( 0·07)	0·52	0·11 (0·01)	0·06 (0·01)	0·07 (0·01)	1·12 (0·02)

The expected and estimated SNP effects for various binary proportions (Table 6) coincided well (formula for computing expected values in Appendix S1). The power to detect causal SNPs decreases for skewed ratios of 0's to 1's, dropping from 95% to 92% for the simulated scenarios. The inflation factor *λ* was close to 1·00 for all proportions. The model therefore seems to be suitable for binary data, but the power to detect causal SNPs decreases when the proportion of successes for the analysed trait decreases.

**Table 6:** Performance of repeated measurement model for different binary proportions. An additive effect size of 1·0 for one QTL was simulated on the underlying Gaussian scale

Binary cut off	Proportion 1's	Expected estimate^a^	Estimated effect size	Prop. $P<10^{-6}$	*λ*
0	0·63	0·201	0·202 (0·028)	0·95	1·02 (0·02)
−0·5	0·73	0·172	0·172 (0·026)	0·95	1·01 (0·02)
−0·75	0·77	0·155	0·155 (0·020)	0·93	1·01 (0·03)
−1·0	0·80	0·136	0·137 (0·022)	0·91	1·01 (0·02)
−1·25	0·84	0·118	0·118 (0·018)	0·92	1·00 (0·02)^b^

a Expected value on the observed binary scale derived in Appendix S1.

b Not estimated for one of the replicates due to non‐convergence using the GenABEL estlambda function.

## Discussion

Here, we present a method for GWAS in populations having repeated measurements that also allows for population family structure. The r package implementation, RepeatABEL, gives a user‐friendly interface for fitting data having repeated observations, and the proposed algorithm is fast, which makes variance component modelling in GWAS feasible. Our simulations showed that in the case of unbalanced data and data where there is between year variation, inclusion of repeated measures in the analysis increases the power of a GWAS to detect causal variants (Fig. 1). It is therefore reasonable that the proposed method will be useful especially in studies of natural populations where large variation in phenotypes between years is common. Although the method assumes that the trait is normally distributed, it seems robust to the use of binary phenotypic data (except in the extreme case of many QTL having large effects). For traits having extreme binary proportions (either <5% or >95%), this method should be used with care and we suggest that the *P*‐values for the most significant SNPs should be recomputed using a model assuming a binomial response.

Our proposed method is a two‐stage method where the distribution of the residuals and random effects are estimated in a preliminary model that does not include the SNP effects. In the second stage, a model including the SNP effect is fitted. Consequently, part of the SNP effects will be captured by the random polygenic effect fitted in the first preliminary model. This is seen in the results of Table 3 as the polygenic variance component is overestimated for large simulated SNP effects, but still the inflation factor *λ* is around 1·0 (except for the extreme case of 20 QTL having large effects). The over‐estimation of polygenic effects does not seem to affect the detection of significant SNP effects in the GWAS. However, if large SNP effects are detected and the focus is on estimating the polygenic variance then one should fit a linear mixed model including both the random polygenic effects and the detected SNPs (having a significant effect) as explanatory variables, which is feasible since this model only needs to be fitted once after performing the GWAS.

In most previous analyses, repeated measurements in natural populations have been treated as nuisance information and the GWAS performed on average individual values. Hence, repeated measures are considered as unnecessary extra information complicating the analysis. By contrast, the RepeatABEL package takes advantage of repeated measurements to increase power and add information and the analysis is rather easy to perform for users acquainted with the R environment and GenABEL (Aulchenko *et al*. 2007b). Our simulation study focused on applications in natural populations, but the RepeatABEL package is also expected to be useful in human studies. Beyene & Hamid (2014) summarized the proposed methods discussed during the Genetic Analysis Workshop 18. Several of these methods included linear mixed models that incorporated genetic relatedness through kinship matrices, but the computational efficiency of the methods was not studied and the aim of that workshop was not to develop new software.

One of the earliest methods proposed for GWAS having related individuals was GRAMMAR (Aulchenko, De Koning & Haley 2007a). It takes the estimated residuals from a linear mixed model, without SNP effects, and uses these as response in a second linear model including the SNP effect. Extending GRAMMAR to include repeated measurements within the GenABEL framework would have been technically difficult to implement due to the structure of gwaa.data objects in GenABEL. Furthermore, GRAMMAR does not perform as well as the method implemented in RepeatABEL for models including several fixed effects (see Appendix S2), which is highly unsatisfactory especially in ecological field studies where modelling of non‐genetic effects is essential.

A couple of methods have been developed recently for GWAS having repeated observations from unrelated individuals. The GEE method proposed by Sitlani *et al*. (2015) does not include explicit modelling of random effects and requires strong assumptions of the missing data process as the GEE method is not likelihood based. Sikorska *et al*. (2013) proposed a two‐stage method to fit SNP effects for random slopes where individual slope effects are fitted using estimated random effects from a preliminary model without SNP effects. These methods are not suitable for analyses of populations having related individuals. The GEE method does not allow explicit modelling of the genetic relationship between individuals, and two‐stage methods using pre‐computed random effects (i.e. BLUPs) are known to reduce power for populations having related individuals (Ekine *et al*. 2013) unless uncertainty can be carried over with the BLUP estimates (Postma 2006; Valdar *et al*. 2009; Hadfield *et al*. 2010). Our proposed method is also a two‐step approach, because we first estimate the covariance structure without fixed SNP effects and thereafter fit each SNP effect in a generalized least squares given the estimated covariance structure. With our approach, however, the uncertainty in the BLUPs is included in the fitting of the SNP effects because the BLUPs are not pre‐computed but rather estimated separately for each SNP. The proposed method should therefore be relevant and useful for GWAS in populations having repeated measurements on related individuals.

In our simulation study, we found that the crude method of using average individual phenotypic values as response in a GWAS works quite well as long as there are no substantial yearly phenotypic variation and random polygenic effects are included in the model. A possible explanation to this rather surprising result is that the individuals in our simulations are moderately related, and the genomic relationship matrix therefore resembles an identity matrix to some extent. Consequently, the random polygenic effects pick up most of the permanent environmental effects too, when a model using average phenotypes as response is used and random polygenic effects are included in the model. However, long‐term natural studies regularly report significant year effects, ranging from explaining only 2% of the variation to more than 30% (e.g. Qvarnström, Brommer & Gustafsson 2006; Stopher *et al*. 2012; Petelle, Martin & Blumstein 2015). Whilst some studies may not find strong year effects, without testing for these effects explicitly, it is impossible to know how impactful they will be in a GWAS. Furthermore, the advantage of using our proposed repeated measurements model should be greater in populations having a higher degree of relatedness between individuals. In general, one must evaluate the fit of data to the model assumptions on a case‐by‐case basis; thus, the suitability of any model will be population specific.

The repeated measurements model implemented in the RepeatABEL package has a moderate but substantial effect on the power to detect QTL in GWAS of natural populations. The benefit of the method mainly depends on the variance of temporal effects, such as year and age effects, and allows for the estimate of these effects in a GWAS framework. We believe that this development will lead to new findings in GWAS and an increased understanding of evolutionary ecology. Furthermore, understanding evolution driven by natural selection requires studies of wild populations, and with the ever‐cheapening of sequencing, the precision of these studies is increasing. Posing and answering relevant questions in evolutionary biology, including those related to populations' ability to adapt and persist in changing environments, as well as basic research questions about life‐history processes such as reproduction and senescence, require that we ensure that the methods we use suit the data we have.

## Supporting information


**Appendix S1.** Transforming underlying effects to observed effects for binary traits.

**Appendix S2.** RepeatABEL vs. GRAMMAR.
