# Supplementary material for: Increasing the power of genome wide association studies in natural populations using repeated measures – evaluation and implementation
Source: Methods Ecol Evol. 2016 Feb 5;7(7):792–9. doi: 10.1111/2041-210X.12535 (PMC4950150; doi:10.1111/2041-210X.12535)
Supplement: Supplementary file 1 — Appendix S1. Transforming underlying effects to observed effects for binary traits. [file MEE3-7-792-s001.pdf]

Transforming underlying effects to observed effects for binary traits

The developed method assumes that the analyzed trait follows a Gaussian distribution and the possibility to use the method on a binary trait is investigated here. To be able to assess the performance of the estimation method, the expected effects on the observed binary scale need to be derived from the simulated effects on the underlying scale.

A simulated QTL having an additive effect of b_u on the underlying scale (y_u) will have an effect b on the observed trait that depends on the simulated variance components, the threshold τ and the minor allele frequency q. The expected effect on the observed scale is

$$b = (1 - 2q)P_1 + qP_2 - pP_0$$

where $p = 1 - q$ and $P_k = 1 - \Phi\left(\frac{\tau - kb_u}{\sigma}\right)$, with k=0, 1 or 2. Here Φ is the cumulative probability function for a standard normal distribution and $\sigma^2 = \sigma_g^2 + \sigma_p^2 + \sigma_e^2$.

The expected variance components on the observed scale can be computed from the variance components on the underlying scale following Lee et al. [2011]. For instance, the total observed residual variance is 0.25 for $\tau = 0$ (since the binomial proportion is 0.5) and the heritability on the observed scale is $4h^2\phi(0)^2$ where h^2 is the heritability on the underlying scale and $\phi()$ is the standard normal density function. For $h^2 = 0.33$ we then get a heritability on the observed scale of 0.21 and an expected genetic variance of 0.053.

References

S. H. Lee, N. R. Wray, M. E. Goddard, and P. M. Visscher. Estimating missing heritability for disease from genome-wide association studies. *The American Journal of Human Genetics*, 88(3):294–305, 2011.
