# Supplementary material for: Increasing the power of genome wide association studies in natural populations using repeated measures – evaluation and implementation
Source: Methods Ecol Evol. 2016 Feb 5;7(7):792–9. doi: 10.1111/2041-210X.12535 (PMC4950150; doi:10.1111/2041-210X.12535)
Supplement: Supplementary file 2 — Appendix S2. RepeatABEL vs. GRAMMAR. [file MEE3-7-792-s002.pdf]

# RepeatABEL versus GRAMMAR

Here the RepeatABEL approach is compared to GRAMMAR. Both of these are two-step approaches but they are fundamentally different. In a preliminary model without SNP effects RepeatABEL fits a distribution for the residuals and random effects, whereas GRAMMAR fits point estimates of the residuals and random effects.

In real-life applications it is reasonable to expect both methods to give similar results as long as the model is simple with few explanatory variables. However, this is not the case for more advanced models. In the example below, it is shown that RepeatABEL will outperform GRAMMAR rather dramatically when the number of fixed effects increases (Figure S1).

The example simulates 100 observations from 100 unrelated individuals (i.e. observations can be treated as independent). A minor allele frequency of 10% is simulated and the additive SNP effect (for each minor allele) is 2 phenotypic standard deviations. The number of fixed effects included in the model were varied from 1 to 50. 1000 simulation replicates were used for each case.

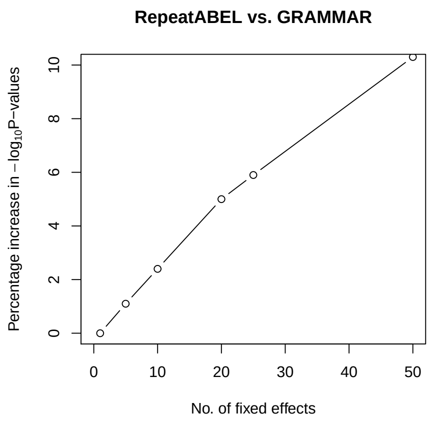


Figure S1: Expected percentage increase in -logP-values for RepeatABEL compared to GRAMMAR at a simulated SNP locus. 100 observations from unrelated individuals simulated using 1000 simulation replicates per scenario where the number of fixed effects is varied between scenarios going from 1 up to 50.

### Summary of GRAMMAR and RepeatABEL two-step algorithms

Here, the two-step approaches of GRAMMAR and RepeatABEL are summarized in a bit more technical detail to highlight their differences.

The two-step GRAMMAR approach is conducted as follows:

1. Fit a linear model with all explanatory variables included except the SNP effect, and compute the residuals.

2. Use the residuals as response in a second linear model including the SNP effect.

The two-step RepeatABLE approach is conducted as follows:

1. Fit a linear model with all explanatory variables included except the SNP effect, and estimate the variance matrix, $V$, for the random effects and residuals.

2. In a second linear model, include the SNP effect as explanatory variable where the model is constrained to have a variance matrix proportional to $\hat{V}$.

### R code used for simulating the case including 50 fixed effects

```
> #A simple, but extreme case, showing
> #the advantage of RepeatABEL compared to a GRAMMAR approach.
> #Note that no approximation for RepeatABEL needed in this example,
> #because the observations are iid.
> set.seed(1234)
> n = 100 #No. of individuals
> p = 50 #No. of class effects, "groups"
> grp <- as.factor(rep(1:p,each=2)) #Group factor
> b = 2.0 #SNP effect
> n.rep = 1000 #No. of simulation replicates
> pGRAMMAR <- pRepeatABEL <- numeric(n.rep)
> for (i.rep in 1:n.rep) {
+   x <- rbinom(n, 2, 0.1) # 10% MAF
+   y <- b*x + rnorm(n)
+   lm0 <- lm(y ~ grp)
+         lm01 <- lm(lm0$res ~ x)
+         lm1 <- lm(y ~ grp + x)
+         pGRAMMAR[i.rep] <- summary(lm01)$coef[2,4]
+         pRepeatABEL[i.rep] <- summary(lm1)$coef[p+1,4]
+ }
> ######### RESULTS ######
> logP_RepeatABEL <- -log10(pRepeatABEL) #log P values from RepeatABEL
> logP_GRAMMAR <- -log10(pGRAMMAR) #log P values using GRAMMAR
> #10.3% expected increase in -log10P-values using
```

```
> #the RepeatABEL approach compared to GRAMMAR
> cat( "Percentage increase:",
+      round( 100*(lm(logP_RepeatABEL ~ 0 + logP_GRAMMAR)$coef - 1), 3 ) )

Percentage increase: 10.26
```
